# The potential function of soy protein hydrolysate to induce myogenic differentiation of C2C12 cells

**DOI:** 10.1371/journal.pone.0321650

**Published:** 2025-04-16

**Authors:** Yinglei Chen, Changwu Xiong, Yingzhi Wan, Mengjun Sun, Zhong Zheng, Dayou Liu, Huilin Liao, Yueqing Wang, Yexu Wu

**Affiliations:** 1 Hubei Provincial Key Laboratory of Yeast Function, Angel Yeast Co., Ltd., Yichang, China; 2 Hubei Key Laboratory of Tumor Microenvironment and Immunotherapy (School of Basic Medicine, China Three Gorges University), Yichang, China; 3 EncoreBio Co., Ltd., Hangzhou, China; Università degli Studi della Campania, ITALY

## Abstract

Muscle satellite cell (MSC) isolation, proliferation, and differentiation are the basis of cultured meat (CM) technology, which emerged as a sustainable and moral substitute for conventional animal agriculture. Notwithstanding the encouraging future of CM, there are still a lot of obstacles to overcome, like the high expense of cell culture media and the need for fetal bovine serum (FBS). The goal of this work is to determine whether plant-based nitrogen source soy protein hydrolysate (SPH) can improve myogenic differentiation and functional development in MSCs cultured for CM by acting as a serum substitute. We concentrated on how Angel Yeast Company’s SPH PU041 affected the C2C12 mouse cell line, a useful model for studying muscle biology and the CM sector. Adding PU041 to cell culture media containing different concentrations of FBS was found to promote C2C12 cell proliferation and elongation, with optimal effects observed at 0.5 g/L. Immunofluorescence and flow cytometry analyses revealed that PU041 up-regulated the protein levels of myosin heavy chain (MyHC) and myogenic differentiation factor 1 (MyoD), key biomarkers in myogenesis. Furthermore, quantitative real-time PCR (qPCR) confirmed the up-regulation of MyHC, MyoD, and myogenin (MyoG) mRNA expression, indicating that PU041 induces myogenic differentiation. The findings suggest that SPH PU041 can potentially be used to reduce the costs associated with CM production as a viable serum substitute, thereby facilitating a more sustainable and ethical approach to food production. However, the precise mechanisms underlying PU041’s effects on myogenic differentiation warrant further investigation.

## Introduction

Muscle satellite cells (MSCs) cultivated *in vivo* are the basis for the novel technique known as “cultivated meat” (CM), which is used to produce edible tissue [1,2]. Currently, CM is approved for sale and consumption in the USA, Israel, and Singapore. This illustrates how an idea from the future has progressively become the current reality [3]. Although there are numerous challenges in the way of this evolution, which offers a far more moral and environmentally friendly substitute for conventional animal husbandry, one of the biggest is the high expense of large-scale culture medium, which is mostly concentrated on the use of fetal bovine serum (FBS) for culture [4]. In the early, serum is present in almost all cell culture media. Because FBS contains a variety of growth factors, mainly as nutrients, hormones, and growth factors, that are frequently required for cell growth, it is essential for cell culture. Unfortunately, using serum comes with a significant cost and raises ethical questions regarding its manufacture [5]. For example, adding serum to the medium may bring exogenous pollution, such as animal-derived viruses and mycoplasma [6,7]. In addition, serum also has many problems in industrial production, such as downstream process, product purification, variable raw materials, limited product supply and high cost, which makes people always explore alternative products for serum [8]. Therefore, researching serum-free media and looking for alternatives to FBS in MSC culture are crucial steps in the development of CM.

In 1972, Taylor began to study the replacement of serum with protein hydrolysates in mammalian cell cultures [9,10]. As research into the factors influencing animal cell growth progresses, the application of protein hydrolysates in cell culture has become increasingly widespread. However, the addition of animal-derived protein hydrolysates to human injectable biopharmaceuticals poses safety concerns, hence the production process generally favors the use of plant-derived protein hydrolysates [11].

Soybean contains nearly 40% protein and its amino acid composition is essentially identical to that of animal proteins.Thus, the process of enzymatic hydrolysis, separation and extraction, filtration, evaporation, and spray drying results in the mixture of oligopeptides, polypeptides, and amino acids known as soy protein hydrolysate (SPH), becomes the only plant protein capable of replacing serum [12]. SPH has good solubility under conditions of significant changes in pH, temperature, ionic strength, and ammonia concentration, which is one of the most important physicochemical properties. Furthermore, it has been discovered that SPH exhibits a range of biological activities, including antioxidant properties, the capacity to lower blood lipids and cholesterol levels, and hypoallergenic characteristics, among other functions [13]. Nowadays, SPH has already been employed in bacterial and cell cultures as a nitrogen-rich source [14,15]. But no one has ever investigated SPH’s role in CM culture. Soy protein, being a plant-based source of nitrogen, offers clear and substantial benefits when it comes to being environmentally friendly and renewable when used to create culture media [16]. Furthermore, the expense of large-scale CM culture would be significantly decreased if SPH could be a viable serum replacement.

MSCs often have a limited ability to differentiate into distinct types of cells. Finding the right supplements to improve myogenic differentiation and functional development in systems of cell culture is crucial. The myogenic differentiation factor 1 (MyoD) was identified as a member of the myogenic regulatory factors (MRFs). It has been demonstrated that overexpressing of MyoD will cause fibroblasts in culture to become skeletal myocytes [17]. One particular muscle filament maturation marker protein that is expressed in mature myotubes is the myosin heavy chain (MyHC) [18]. MyoD and MyHC are therefore regarded as significant regulatory elements and indicators during the activation of MSCs at various phases of differentiation and myogenesis of skeletal muscle cells [19–21].

To better understand the potential of SPH of Angel Yeast Company’s production PU041, we looked at its ability to promote cell growth and induce myogenic differentiation in mouse C2C12 cells—a useful research cell line for the field of muscle biology and the CM industry.

## Methods and materials

### Cells and Cell culture

Mouse C2C12 (RRID: CVCL_0188) were kindly provided by Pricella Life Science & Technology Co.,Ltd. This cell line is cultured with DMEM (DuIbecco’s modified eagIe’s medium) (Gibco, USA) containing 10% fetal bovine serum (Gibco, USA) or 2% horse serum (Gibco, USA), maintained in a 5% CO_2_ cell culture incubator at 37°C. PU041 (Soy protein hydrolysate, Angel Yeast Co., LTD.) is dissolved in the medium and filtered by 0.22 µm before. When we got and used cells, they have been detected without the presence of mycoplasma.

### Measurement of cell length

Cells were examined by differential interference contrast (DIC) (Olympus IX73). Then, we used Image J to visualize and quantify the cell length.

### Preparation methods of SPH PU041

Weigh a certain amount of soy protein powder (Angel Yeast Co., LTD.) to prepare a substrate solution with a protein concentration of 100 g/L. Adjust the pH to 7.0 and then add a composite protease (at a dosage of 500 U/g) to hydrolyze for 6 hours. Throughout the hydrolysis process, maintain the pH of the system constant with 1 M NaOH. After the reaction completed, the mixture is inactivated by boiling water bath, cool, and then centrifuge to obtain the supernatant as SPH PU041.

### Total RNA extraction and RT-PCR/qPCR analyses

Quantitative real-time PCR (qPCR) was conducted using a QuantStudio Flex real-time PCR system (Thermo Fisher Scientific, USA) and TransStart Top Green qPCR SuperMix (Transgene Biotech, China). The sequences of the primers used are listed in Table 1. The detailed steps and RNA extraction methods were performed as described previously [22].

**Table 1:** List of primers used for quantification of specific gene expression.

Gene	Forward (5’-3’)	Reverse (5’-3’)
*myoD*	TGAATGAGGCCTTCGAGACG	GCCTGCAGACCTTCGATGTA
*my* ***HC***	CGGTGAAGGGCATGAGGAAGAG	GCGGAACTTGGACAGGTTGGT
*myoG*	AATGCACTGGAGTTCGGTCC	TTCGTCTGGGAAGGCAACAG
***cyclinD1***	CCCTGACACCAATCTCCTCAAC	CGCATGGATGGCACAATCT
***Wnt 3a***	TCCGACTCT TGGCAGAACTT	AATGGAATAGGTCCCGAACA
***β-catenin***	TGCTGAAGGTGCTGTCTGTC	TCGGTAATGTCCTCCCTGTC
***cmyc***	GCTCGTCTCAGAGAAGCTGG	GCTCAGATCCTGCAGGTACAA
***gapdh***	TCTCCTGCGACTTCAACA	TGTAGCCGTATTCATTGTCA

### Immunofluorescent

MyHC was detected using Goat anti-rabbit IgG labeled with TRITC (ZSGB-BIO Company, China). Then cells were collected and conducted as standard procedures described previously for immunofluorescent localization assays [23].

Besides, MyHC and MyoD was detected using Goat anti-rabbit IgG tagged with TRITC (ZSGB-BIO Company, China), respectively. The Mean fluorescence intensity (MFI) of the cells was recorded by flow cytometry (BD, USA).

### Cell proliferation

A total number of 3×10^3^ C2C12 cells were cultured in one well of 96-well plate. Cell proliferation was monitored every 24 hours for 48 hours with CCK8 (Solarbio, China).

### Statistical analyses

All the experiments were conducted independently in triplicate. Statistical analyses were perfomed using Origin and the data present are as average values ± s.d. *P* values were calculated by the Student’s *t*-test and One-way Anova test and *p*<0.05 was considered significant. Data were obtained from three independent experiments and presented as average values ± s.e.m. (*: *p* < 0.05; **: *p* < 0.01; ***: *p* < 0.001).

## Results

### PU041 promotes proliferation of C2C12 cells both under normal and low-serum conditions

To find a serum substitute for C2C12 cells, we screened a variety of Angel Yeast-produced hydrolyzed vegetable protein (HVP), using one SPH product from Becton, Dickinson Company as a reference. These findings demonstrated that, in various serum circumstances, the majority of Angle Yeast had growth promoting effects on C2C12 cells. Particularly, under all serum concentrations, PU041 showed the strongest growth promoting effects under all serum concentrations (Table 2).

**Table 2:** C2C12 cells were cultured for 24 h in the presence of different concentrations of FBS and 0.5 g/L different types of HVPs.

	10% FBS	5% FBS	2% FBS	1% FBS	0% FBS
PU041	+	+++	++	+++	+++
FP400	ns	++	+	+	+
FP402	ns	+	–	+	---
FP410	ns	+++	++	++	--
FP502	--	--	---	---	---
I02	--	--	--	--	--
BD	ns	+	+	ns	--

Each group was compared to the group without HVP addition. ns represents no significance, + represents the up-regulation of. is between 3%-5%, ++ represents the up-regulation of. is between 5%-10%, +++ represents the up-regulation of. is over 10%, - represents the down-regulation of. is between 3%-5%, -- represents the down-regulation of. is between 5%-10%, and --- represents the down-regulation of. is over 10%.

**Fig 1:**
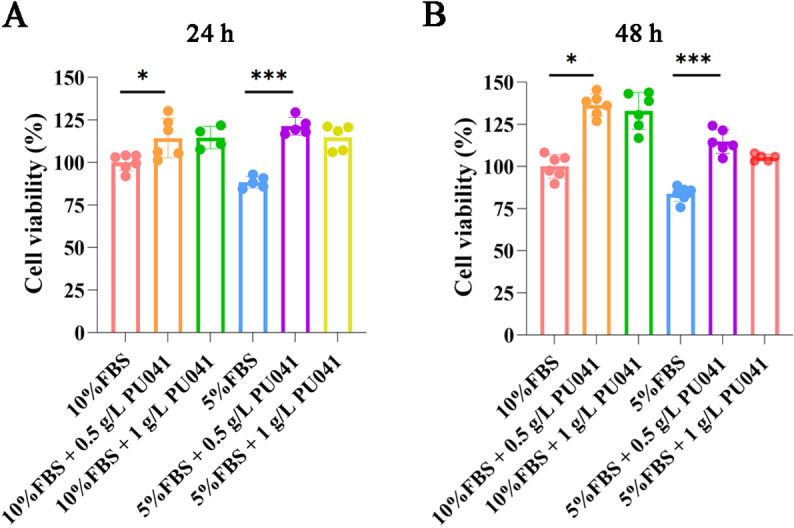
Promoting effect of PU041 on cell growth of C2C12 cells. The cell viability was detected by CCK-8 reagents after C2C12 cells culturing in different conditions for 24 h(A) and 48 h(B), and 10%FBS group was used as a standard to process data. * represents *p*< 0.05, and *** represents ****p**** < 0.001.

The cell growth-promoting effects of PU041 was further evaluated on C2C12 to explore its potential in reducing CM costs as a serum substitute. It was found that PU041 stimulates C2C12 cell proliferation more effectively in cultures with 5% FBS compared to 10% FBS. Additionally, the proliferation effect was more evident over 48 hours than 24 hours. 1 g/L PU041 had no obvious better impact on promoting cell proliferation than 0.5 g/L (Fig 1). We also found the promotion of PU041 on cell growth was not entirely in a dose-dependent manner, for 2 g/L PU041 inhibited cell proliferation. Besides, 0.5 g/L or 1 g/L PU041 had better effect than ITS, an established serum substitute (S1 Fig). Therefore, 0.5g/L PU041 was used in further investigations to explore its potential functions.

### PU041 promoted cell elongation

**Fig 2:**
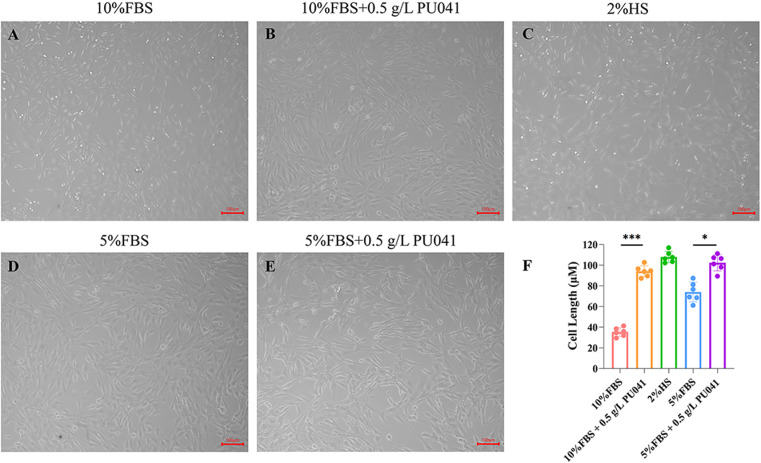
Promoting effect of PU041 on cell length of C2C12 cells. A-E: The cells were cultured in different conditions for 24 h, and visualized by bright field, the scale bar indicates 100 μm; F: The cell length were quantitatively measurd and analyzed. * represents ****p**** < 0.05, and *** represents ****p**** < 0.001.

The differentiation of MSCs is of great importance for CM culture. Traditionally, 2% horse serum (HS) medium was used to induce the differentiation of C2C12 cells. The cell length elongated to 107.93 μm from 38.45 μm as control after adding 2% HS for 2 days, and the cell length of cells extended to 94.02 μm in the culture with 10% FBS and 0.5 g/L PU041 added. Besides, we found PU041 also promoted the cell length elongated to 74.03 μm from 102.28 μm in the culture containing 5% FBS (Fig 2). Thus, we concluded PU041 had promotion effect of cell elongation in the culture containing 10% and 5% FBS.

### PU041 increases the expression of muscle-specific factors related to myogenesis

**Fig 3:**
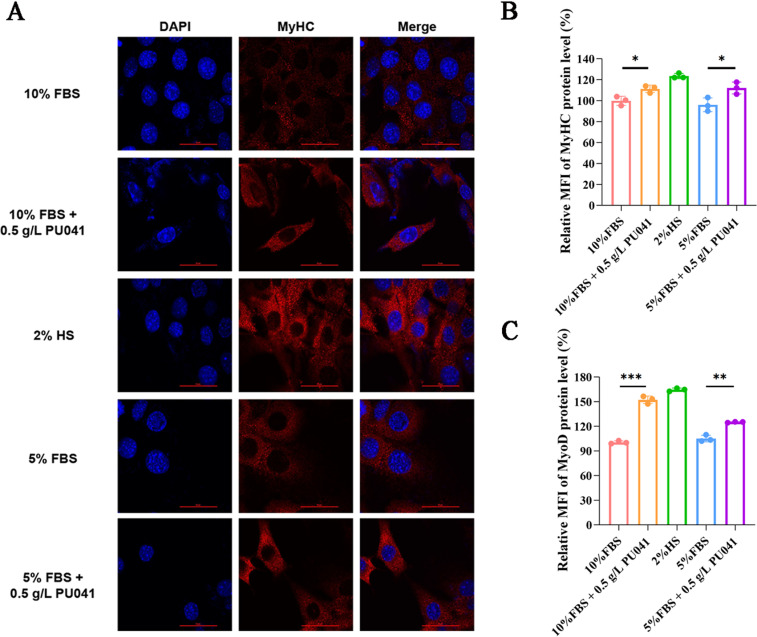
Promoting effect of PU041 on the expression of MyHC and MyoD of C2C12 cells. A: MyHC was detected by TRITC red fluorescence after immunofluorescence after C2C12 cells cultured in different conditions for 24 h; B-C: The MFI of MyHC marked with TRITC red fluorescence after immunofluorescence were quantitatively measurd and analyzed. * represents ****p**** < 0.05, ** represents ****p**** < 0.01 and *** represents ****p**** < 0.001.

To confirm PU041’s effects on C2C12 cell differentiation, immunofluorescence was employed to examine if PU041 influences the expression of MyHC and MyoD, which are crucial for myogenic differentiation. As shown in Fig 3A and 3B, flow cytometry and laser scanning confocal microscopy demonstrated that, after two days, PU041 could up-regulate the protein level of MyHC in comparison to the control group. With the addition of PU041 in 5% and 10% FBS culture, the expression of MyoD was also up regulated, serving as a crucial regulatory biomarker for the early stage of differentiation (Fig 3C).

**Fig 4:**
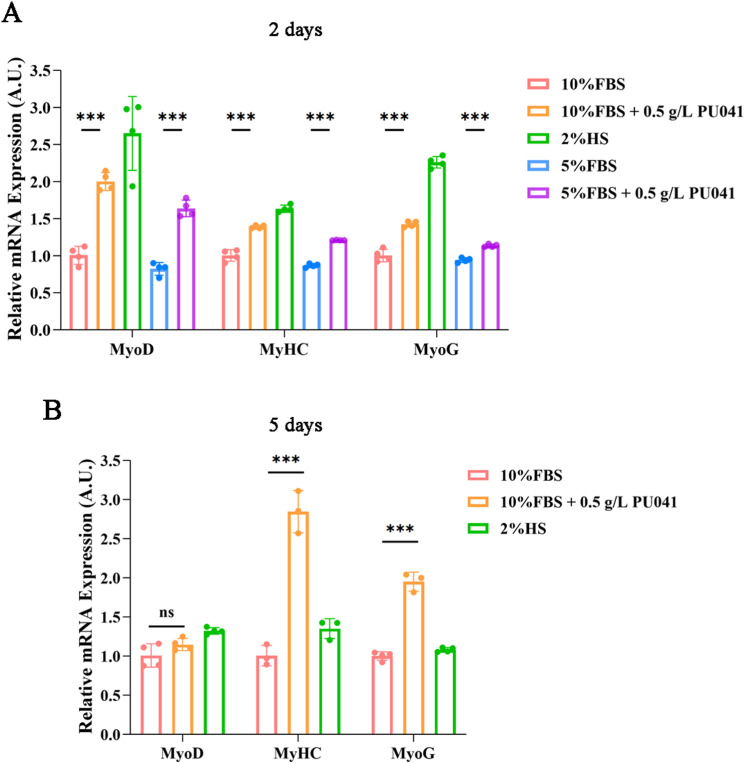
Promoting effect of PU041 on the mRNA expression of genes about myogenic differentiation of C2C12 cells. The expression levels of MyoD, MyHC and MyoG were analyzed by qPCR in C2C12 cells cultured in different conditions for 2 days(A) and 5 days(B). 10%FBS group was used as a standard to process data. ns represents no significance, and *** represents ****p**** < 0.001.

These findings were further corroborated by the RT-qPCR data. PU041 was found to enhance the mRNA expression of MyHC, MyoD, and MyoG in the two-day culture when compared to control groups With just 5% or 10% FBS (Fig 4A). Additionally, it was also found that adding PU041–10% FBS in the five-day culture clearly increased the mRNA expression of MyHC, MyoD, and MyoG (Fig 4B). Overall, these findings demonstrated that PU041 might induce myogenic differentiation.

## Discussion

CM technology is expected to solve some social problems, such as resource shortages and environmental pollution [24]. However, high cost of cell culture media has become an obstacle of CM industry development [25]. Thus, it is of vital importance to search for low-cost substitute to serum.

SPH has been reported to contribute to cell growth frequently [12,13]. BSPH produced by different manufacturing processes may have different effects on cell culture performance, such as the yield of recombinant proteins [18,26]. There Was no report about the effects of SPH on the culture of MSCs before. The present study explores the potential of soy protein hydrolysate (SPH), specifically the PU041 product from Angel Yeast Company, as a serum substitute in the culture of C2C12 cells, which are pivotal in the field of cultured meat (CM) production. Our findings contribute significantly to the ongoing efforts to develop sustainable and ethical alternatives to traditional animal agriculture.

Our results demonstrate that SPH PU041 from Angel Yeast Company effectively promotes the proliferation of C2C12 cells under both normal-serum and lower-serum conditions, suggesting its potential as a cost-effective supplement in CM culture media. This is particularly relevant given the high costs associated with fetal bovine serum (FBS), which has been a significant barrier to the scalability of CM production [4,5]. With the extension of culture time, lower-serum condition could significantly inhibit normal cell growth. But with the addition of PU041, the cell growth rate of C2C12 could be restored. Compared with other serum substitutes, edible SPH have higher safety in CM industry. Besides, according to our research, the cost of 10% FBS culture medium is about $150 per liter currently. Besides, the cost of commercial serum substitute is about $150 per liter. Thus, as our analysis, if 5% FBS is replaced with 0.5g/L PU041, the cost will be reduced by 40%. In any case, SPH PU041 has the potential to be an alternative to serum to reduce the cost of CM production.

We also observed that PU041 not only supports cell proliferation but also enhances cell elongation, a hallmark in the differentiation of muscle satellite cells (MSCs). Because the muscle fiber differentiation efficiency of CM is still very low, Many research has been focused on the structures and mechanisms leading to myogenic differentiation of skeletal muscle cells [27–31]. The biomarkers and mechanisms of C2C12 in myogenesis has been elucidated now. Thus, we not only observed the microscopic phenotype of PU041-treated cells, but also measured the expression of biomarkers of myogenic differentiation using different methods. All these results proved that PU041 could promote myogenic differentiation under normal cell culture conditionswith 10% or 5% FBS for the first 2 days, and further confirmed by the RT-qPCR technology after cultured 5 days with PU041 adding with 10% FBS. This dual functionality underscores the multifaceted role that SPH could play in CM culture, potentially streamlining the process by combining proliferation and differentiation stages.

Moreover, our immunofluorescence and RT-qPCR data revealed that PU041 upregulates the expression of key myogenic regulatory factors, including MyoD, MyHC, and MyoG. These findings are consistent with previous studies highlighting the importance of these factors in skeletal muscle cell differentiation and myogenesis [9,10]. The ability of PU041 to induce myogenic differentiation is a groundbreaking discovery, as it addresses one of the major challenges in CM technology—the low efficiency of muscle fiber differentiation.

The implications of our findings are manifold. By replacing FBS with SPH, we can envision a more sustainable and ethical CM production process. The renewability and eco-friendliness of plant-based nitrogen sources, as demonstrated by SPH, align with the environmental goals of the CM industry [11].

Understanding these mechanisms could lead to the optimization of SPH formulations for CM culture. Previous studies have shown that myogenic proliferation and differentiation of skeletal muscle cells are regulated by the classical Wnt pathway [32,33]. Thus, we further detected and found that 0.5g/L PU041 can upregulated the mRNA expression level of *β-catenin* (1.7 fold, *p*<0.001), *Wnt3a* (1.4 fold, *p*<0.001), *Cyclin D1* (1.3 fold, *p*<0.05) and *cMyc* (1.5 fold, *p*<0.001) for (S2 Fig). However, while our study presents promising results, further research is warranted to elucidate the mechanisms by which PU041 induces myogenic differentiation. The long-term studies are necessary to assess the safety and efficacy of SPH in CM production.

In conclusion, our research provides valuable insights into the potential of SPH as a serum substitute in CM culture. The findings pave the way for future studies aimed at reducing the costs and environmental impact of CM production while maintaining ethical standards. As the CM industry continues to evolve, innovations such as the use of SPH are crucial for addressing the global demand for sustainable protein sources.

**Key Points**

1. SPH of Angel Yeast Company’s production PU041 can promote cell growth in mouse C2C12 cells.
2. PU041 can induce myogenic differentiation in mouse C2C12 cells.
3. SPH the potential to reduce the cost of CM.

## Supporting information

S1 Fig Serum substitution effect of PU041 on C2C12 cells. The cell viability was detected by CCK-8 reagents after C2C12 cells culturing in different conditions for 48 h, and 10%FBS group was used as a standard to process data. * represents *p*< 0.05, and *** represents *p* < 0.001.
(TIF)

S2 Fig The effect of PU041 on the mRNA expression of Wnt signal genes on C2C12 cells. The expression levels of Wnt, β-catenin, cMyc and CyclinD1 were analyzed by qPCR in C2C12 cells cultured in different conditions for 2 days. 10%FBS group was used as a standard to process data. * represents *p* < 0.05, and *** represents *p* < 0.001.
(TIF)
