# Supplementary figures and images for: The potential function of soy protein hydrolysate to induce myogenic differentiation of C2C12 cells
Source: PLoS One. 2025 Apr 16;20(4):e0321650. doi: 10.1371/journal.pone.0321650 (PMC12002504; doi:10.1371/journal.pone.0321650)

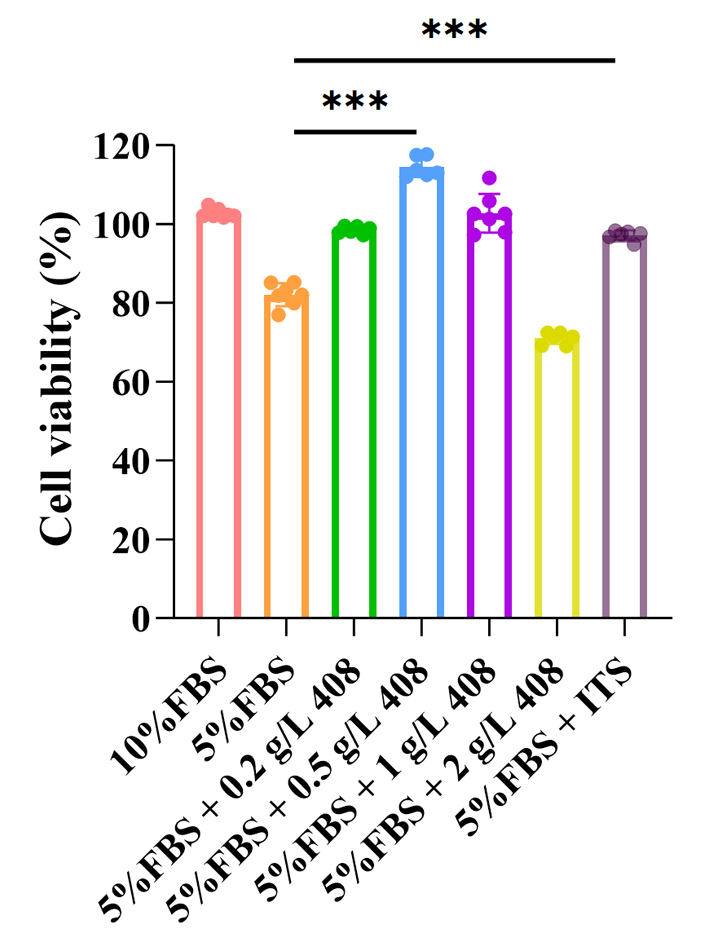

Supplement: S1 Fig — The cell viability was detected by CCK-8 reagents after C2C12 cells culturing in different conditions for 48 h, and 10%FBS group was used as a standard to process data. * represents p< 0.05, and *** represents p < 0.001. (TIF) [file pone.0321650.s001.tif]

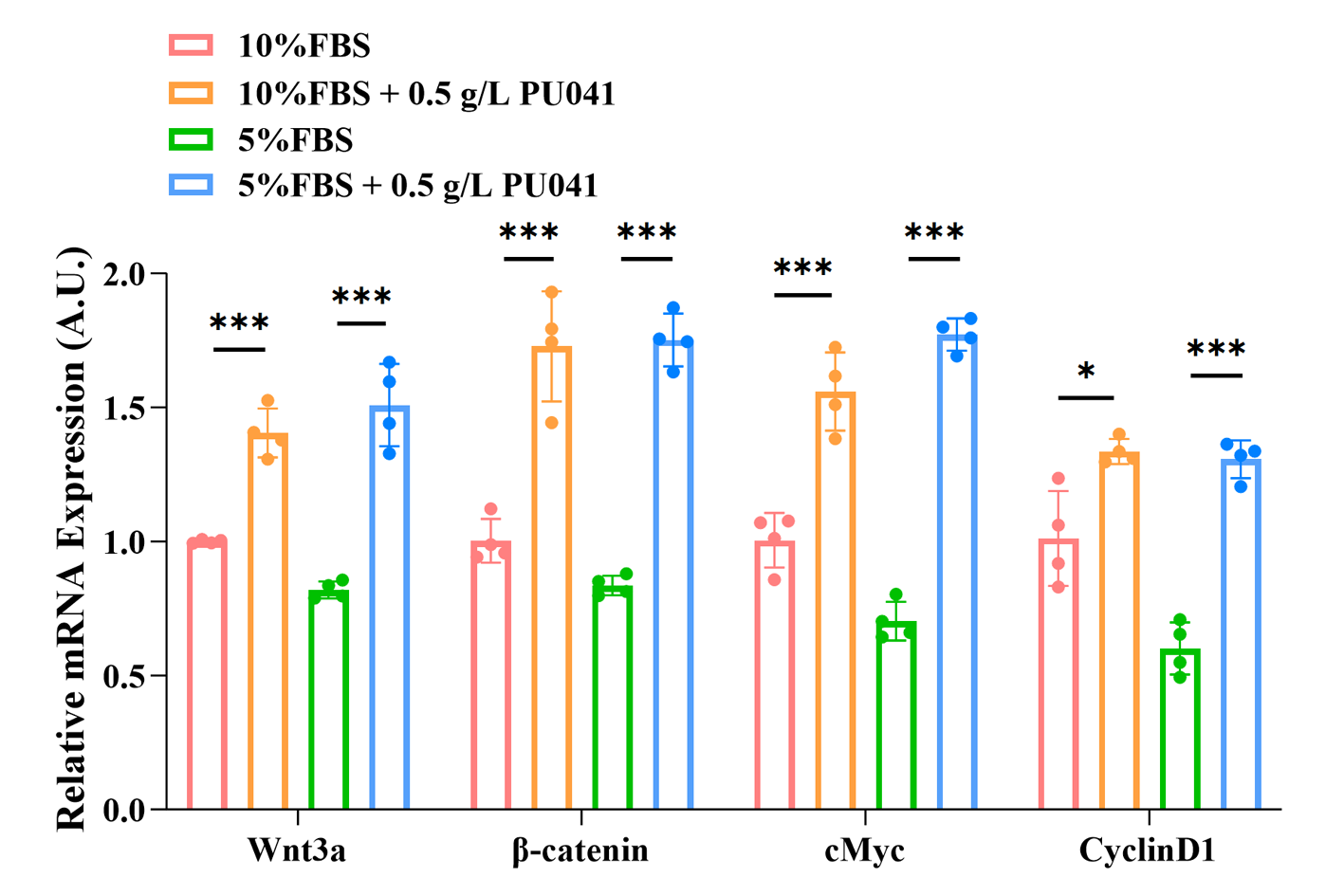

Supplement: S2 Fig — The expression levels of Wnt, β-catenin, cMyc and CyclinD1 were analyzed by qPCR in C2C12 cells cultured in different conditions for 2 days. 10%FBS group was used as a standard to process data. * represents p < 0.05, and *** represents p < 0.001. (TIF) [file pone.0321650.s002.tif]
